# Obtaining artifact-corrected signals in fiber photometry via isosbestic signals, robust regression, and dF/F calculations

**DOI:** 10.1117/1.NPh.12.2.025003

**Published:** 2025-03-31

**Authors:** Luke J. Keevers, Philip Jean-Richard-dit-Bressel

**Affiliations:** University of New South Wales, School of Psychology, Sydney, Australia

**Keywords:** fiber photometry, data analysis, robust regression, isosbestic signals, dF/F

## Abstract

**Significance:**

Fiber photometry is a powerful tool for neuroscience. However, measured biosensor signals are contaminated by various artifacts (photobleaching and movement-related noise) that undermine analysis and interpretation. Currently, no universal pipeline exists to deal with these artifacts.

**Aim:**

We aim to evaluate approaches for obtaining artifact-corrected neural dynamic signals from fiber photometry data and provide recommendations for photometry analysis pipelines.

**Approach:**

Using simulated and real photometry data, we tested the effects of three key analytical decisions: choice of regression for fitting isosbestic control signals onto experimental signals [ordinary least squares (OLS) versus iteratively reweighted least squares (IRLS)], low-pass filtering, and dF/F versus dF calculations.

**Results:**

IRLS surpassed OLS regression for fitting isosbestic control signals to experimental signals. We also demonstrate the efficacy of low-pass filtering signals and baseline normalization via dF/F calculations.

**Conclusions:**

We conclude that artifact-correcting experimental signals via low-pass filter, IRLS regression, and dF/F calculations is a superior approach to commonly used alternatives. We suggest these as a new standard for preprocessing signals across photometry analysis pipelines.

## 1 Introduction

Fiber photometry is an increasingly popular neuroscience technique that combines genetically encoded fluorescent biosensor and implanted optic fiber to measure neural population dynamics *in vivo*.^1^^–^^3^ By measuring fluorescence emitted by biosensors, fiber photometry provides insights into biologically relevant processes such as intracellular calcium (${\mathrm{Ca}}^{2+}$; proxy for neural activity) or neurotransmitter release within specific brain regions.

**Fig. 1:**
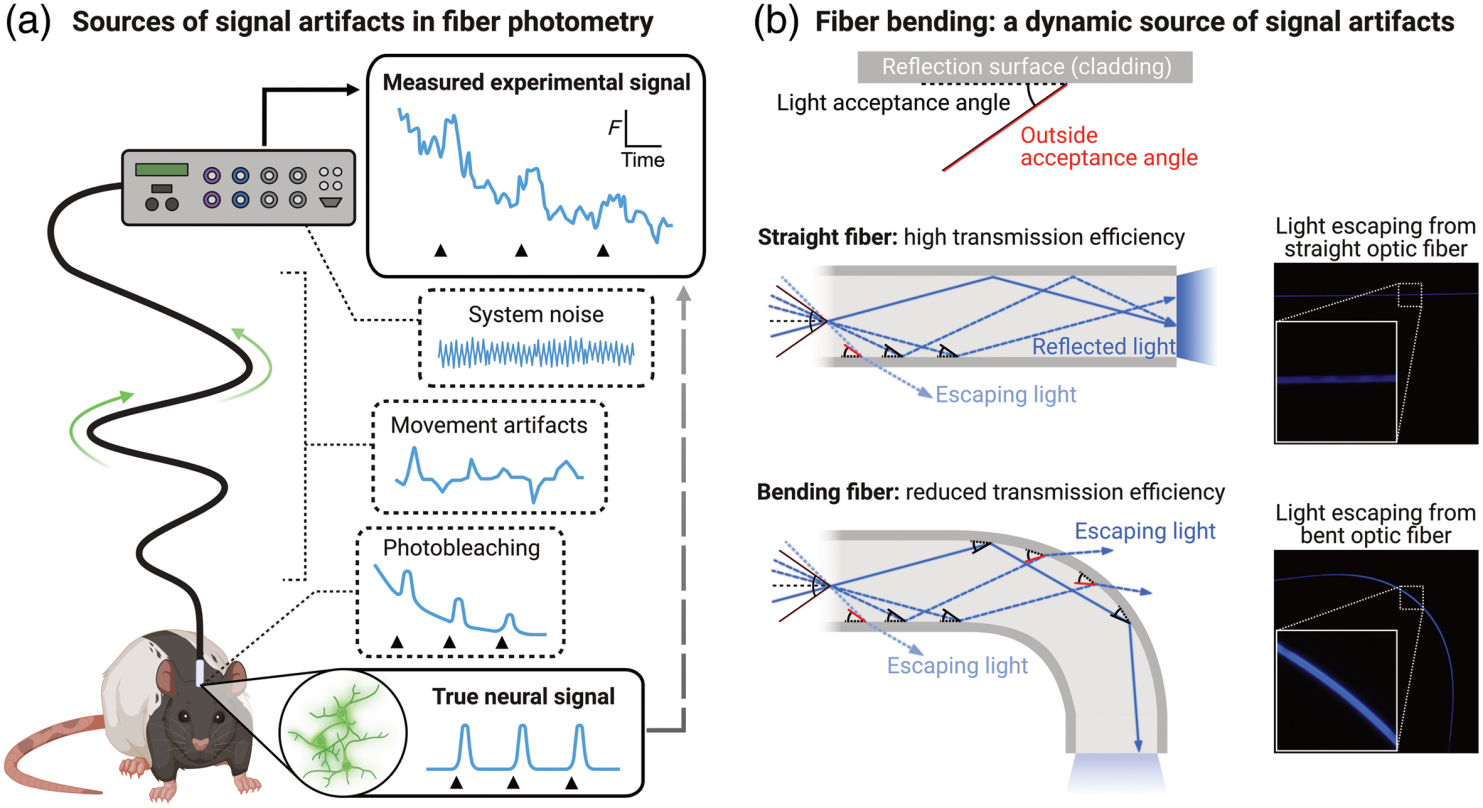
Signal artifacts in fiber photometry. (a) Illustration of artifact sources in fiber photometry experiments. Artifacts include biosensor photobleaching, movement-related artifacts, and system noise (e.g., electrical hum), which contaminate measured signals, obscuring the true neural signal being measured. (b) Fiber patchcord bending is a dynamic source of signal artifacts. Light transmission through a fiber depends upon light within the core being reflected by the outer cladding. This relies on the light striking the cladding within the acceptance angle. Bending in the fiber changes the acceptance angles relative to incoming light rays; more multimodal light is likely to escape the fiber (i.e., not be transmitted) through a bending portion of fiber (same incoming light rays depicted in top and bottom diagrams). Insets: pictures of straight and bending fiber (all other parameters equal); the bending fiber glows brighter (i.e., has more light escaping it) than the straight optic fiber (see also Fig. S1 in the Supplementary Material).

A critical challenge is that fluorescence signals obtained via fiber photometry contain artifacts that undermine the detection and interpretation of signal changes [Fig. 1(a)]. These artifacts include the following:

1. System noise. A prevalent source of noise in photometry recordings is noise from electric components of the photometry system.
2. Photobleaching. Photobleaching occurs when a fluorophore is exposed to excitation light, causing it to output less fluorescence.^4^^,^^5^ Given that photometry requires exposing the biosensor to excitation light across a recording session, this results in artifactual decay in the biosensor signal across the session. Autofluorescence from brain tissue and optical components of the photometry system (e.g., patchcord), which theoretically contributes to an activity-independent baseline to recorded signals, is also subject to photobleaching and thus also contributes to decay in the signal baseline.^1^
3. Motion-related artifacts. An especially problematic source of artifacts stems from animals’ movements. Movement causes the optic fiber patchcord, which relays excitation light and emission fluorescence between the animal and photometry system, to bend. This bending reduces light transmission through the fiber^6^^,^^7^ [Fig. 1(b) and Fig. S1 in the Supplementary Material]. The effect of fiber bending on measured signals is dynamic and continuous and can correlate with an animal’s location and behavior. This complicates the interpretation of location- or behavior-related signals (key-dependent variable in many photometry studies) as signal changes may reflect neural dynamics and/or fiber bending.
4. Hemodynamic artifacts. Blood flow and blood oxygenation can also dynamically attenuate recorded signals via light absorption.^8^ Like movement-related artifacts, hemodynamic artifacts can correlate with behavior.

**Fig. 2:**
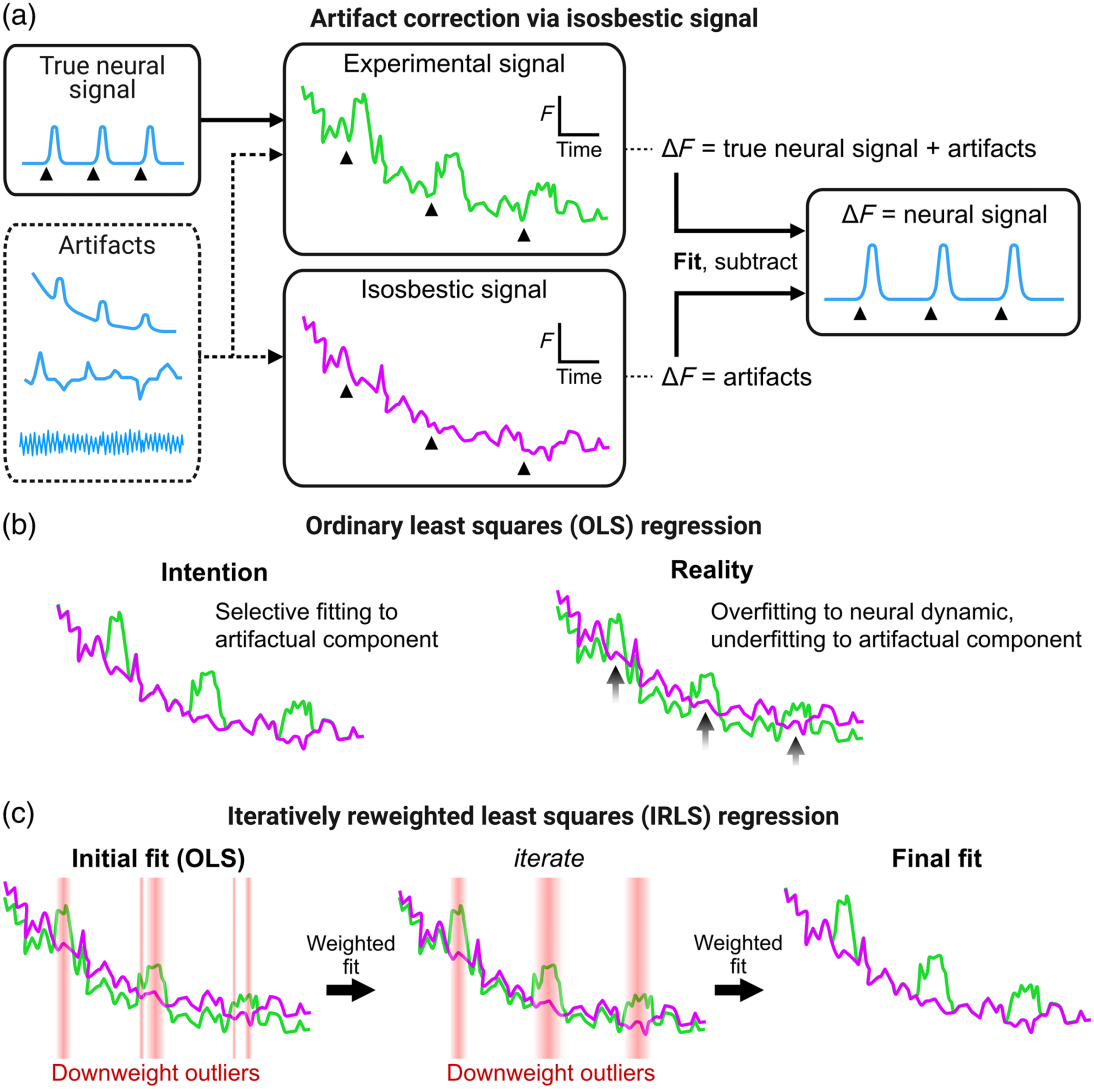
Concept for artifact correction using an isosbestic signal. (a) The experimental signal is composed of both neural dynamic and artifactual components, whereas the isosbestic signal is only composed of artifactual components. In theory, the neural dynamic component can be isolated by fitting and subtracting the isosbestic signal from the experimental signal. (b) Fitting the isosbestic signal to the experimental signal is often done via linear OLS regression. An issue with this approach is that experimental and isosbestic signals, by design, diverge from each other according to neural dynamics. OLS does not partition this, resulting in overfitting of the isosbestic signal to the neural dynamic component of the experimental signal, while underfitting to the target artifactual component. (c) A solution to this is IRLS, a form of robust regression that iteratively downweights poorly predicted datapoints (e.g., times when neural dynamics are occurring) to focus the fit onto the shared artifactual component.

A number of solutions to mitigate many of these artifacts have existed since the earliest fiber photometry studies.^1^^,^^3^ A common preprocessing step to reduce signal contamination by system noise is to apply a low-pass filter to recorded signals. This is because biosensor kinetics typically operate on slower (subsecond, but not microsecond) timescales relative to higher frequency electrical noise components. Signal decay from photobleaching is partially mitigated using low-intensity excitation light but is also dealt with through statistical procedures such as dF/F calculations (discussed below). Movement-related artifacts can be reduced by optimizing the recording set-up, but this problematic source of artifacts requires a direct address. An elegant solution to counteract movement and hemodynamic artifacts is to concurrently measure an isosbestic control signal, which does not fluctuate with neural dynamics but does track artifacts [Fig. 2(a)], to remove the artifactual component of the neural dynamic-tracking experimental signal.^3^^,^^8^

The isosbestic signal of a biosensor works on the principle that the coupling between a neural dynamic and biosensor fluorescence depends on the wavelength of excitation light used to elicit fluorescence. For example, green fluorescence emitted by ${\mathrm{Ca}}^{2+}$ indicator GCaMP in response to blue (∼460 to 490 nm) excitation light is highly ${\mathrm{Ca}}^{2+}$-dependent, but its fluorescence in response to ∼410  nm violet light (isosbestic point) is ${\mathrm{Ca}}^{2+}$-independent.^3^^,^^9^^,^^10^ Thus, GCaMP fluorescence under blue light can serve as the experimental signal to indicate levels of ${\mathrm{Ca}}^{2+}$, albeit with contamination from artifacts, whereas GCaMP fluorescence under violet light can serve as an isosbestic signal that only tracks artifacts. With the correct statistical procedure, the isosbestic signal can be used to remove the artifactual component of the experimental signal to obtain a truer readout of the dynamic being studied (e.g., intracellular ${\mathrm{Ca}}^{2+}$ in GCaMP studies) [Fig. 2(a)]. The same logic applies if a spectrally distinct, static fluorophore (e.g., red-fluorescent tdTomato) is used as the artifact-tracking control signal.^1^^,^^8^ Control signals from a biosensor’s isosbestic point or a static fluorophore are logically interchangeable in the current study.

There are several methods by which the isosbestic signal has been used to correct the experimental signal across studies.^1^^,^^11^^,^^12^ A common way is to fit the isosbestic signal to the experimental signal via ordinary least squares (OLS) regression.^3^ This fitted isosbestic signal is subtracted from the experimental signal and, if the original isosbestic dF/F equation [(experimental signal − fitted isosbestic) / fitted isosbestic]^3^ is followed, is also used to divide the difference signal. The subtraction is designed to remove photobleaching decay and movement-related artifacts from the experimental signal, whereas dividing the isosbestic baseline theoretically counteracts the proportional signal loss due to photobleaching and movement-related artifacts.

A problem with this approach is that OLS regression assumes that all datapoints in the experimental signal should be used to fit the isosbestic signal. This is unsuitable for fiber photometry data because the experimental signal is, by design, expected to diverge from the isosbestic signal in a neural dynamic-related manner. These neural dynamic-related divergences function as outliers in the prediction, which have an outsized influence on OLS-based regression due to these “errors” being squared. This causes the isosbestic signal to be overfitted to the neural dynamic component of the experimental signal while being underfitted to the intended artifactual component [Fig. 2(b)]. This undermines the accuracy of the obtained dF/F and all subsequent analyses.

A solution to the influence of outliers on regressions is a family of approaches known as robust regression. Of these approaches, iteratively reweighted least squares (IRLS) is well suited to addressing the issue of properly fitting isosbestic signals onto experimental signals. In IRLS, each datapoint is weighted between 0 and 1, which determines the degree to which that datapoint influences the regression solution. An initial regression giving all datapoints a weighting of 1 (equivalent to OLS) is conducted. Weightings are then recalculated based on the residual per datapoint and chosen weighting function; outliers are downweighed, and another weighted regression is conducted to obtain a new fit. This is repeated iteratively, with each iteration recalculating weightings and obtaining a weighted fit. The core concept is that this process allows the isosbestic signal to be increasingly fit onto the components of the experimental signal that it strongly predicts (the putative artifactual component of the experimental signal) while disregarding parts of the experimental signal that it does not predict well (the putative neural dynamic component) [Fig. 2(c)]. This makes IRLS conceptually preferable to OLS for the purpose of fitting an isosbestic signal to the experimental signal in fiber photometry. However, no study has directly advocated for or validated this approach, and OLS remains the norm.

Another consequential choice that researchers face is how the fitted isosbestic signal is used to artifact-correct the experimental signal. As previously mentioned, the original dF/F calculation involves dividing the isosbestic signal into baseline-normalized signals.^3^ This theoretically counteracts the proportional loss of signal due to photobleaching and fiber bending across the session. However, not all studies report a “dF/F” divide by the changing isosbestic baseline. Instead, some procedures only subtract the fitted isosbestic from the experimental signal^11^^,^^12^ and are thus better characterized as calculating a dF signal. Although these procedures typically include initial detrending and normalization of experimental and isosbestic signals to remove overall decay trends attributable to photobleaching, these approaches do not counteract putative attenuation of signal dynamics across the session. That is, these procedures address the change in signal baselines but do not address corresponding changes in signal scaling (e.g., its effect on event-related signals) across the session.

## 2 Comparing Artifact Correction Options via Photometry Simulations

**Fig. 3:**
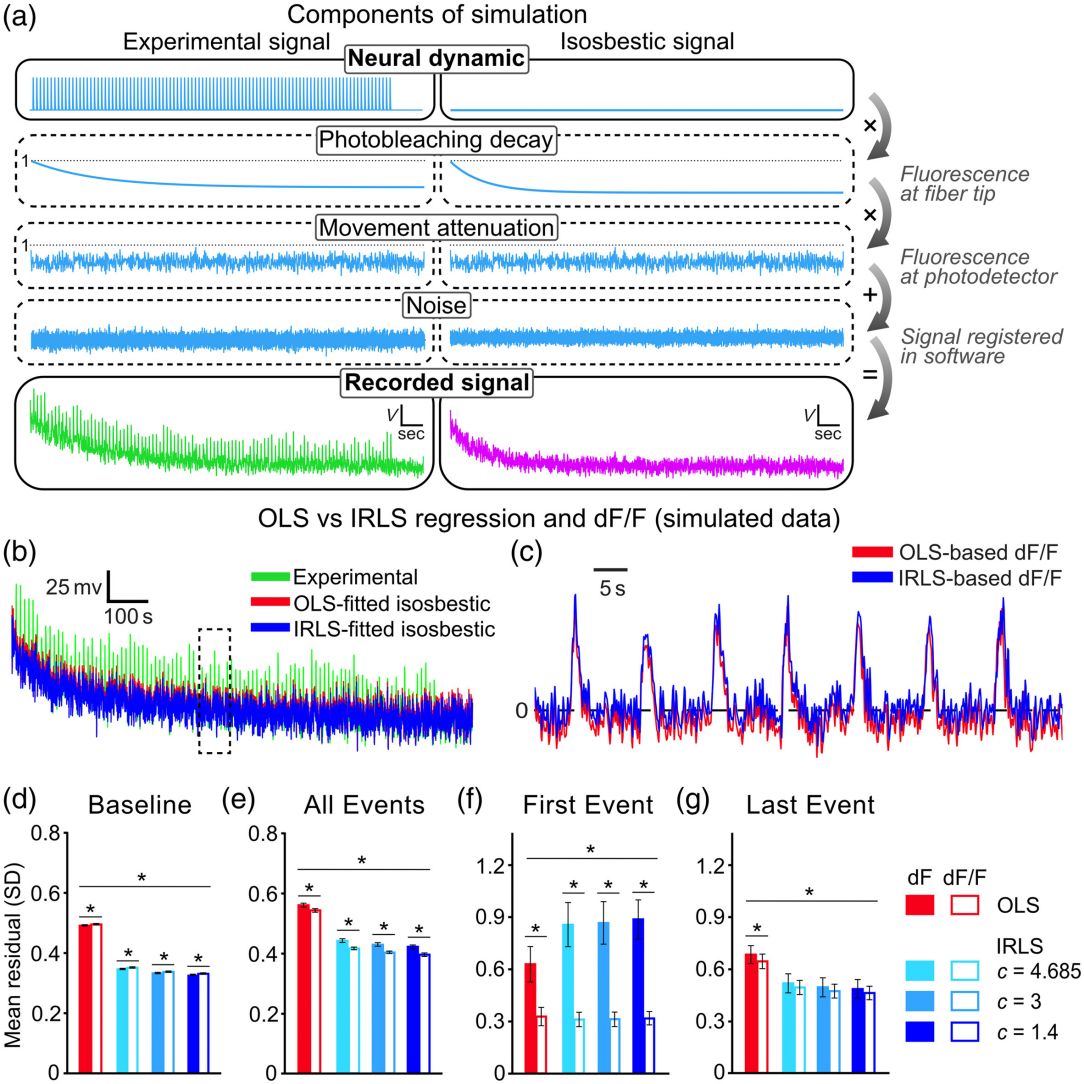
Simulations of fiber photometry signals and analyses. (a) Experimental and isosbestic signal recordings were simulated by applying photobleaching, movement-related signal attenuation, and noise to neural dynamic-dependent versus dynamic-independent signals (100 identical event transients across a 20 min session). (b)–(c) Example fitting of simulated isosbestic onto corresponding experimental signal using OLS versus IRLS regression [(b) whole-session; (c) section indicated by dashed box]. A modest but consequential difference in the baseline is observed. (d)–(g) Mean (±95% CI) absolute residuals during (d) nonevent baseline, (e) across events, (f) first event, and (g) last event, following OLS versus IRLS (c = tuning constant) regressions and dF versus dF/F calculations. Generally, IRLS with lower tuning constants outperformed OLS and IRLS with higher tuning constants, whereas dF/F outperformed dF. *p<0.05.

We empirically tested these different steps for artifact correction using simulated photometry data (Fig. 3 and S3 in the Supplementary Material) modeled after real photometry data.^13^^,^^14^ Simulations were used because the true parameters (e.g., underlying neural dynamics) are known, unlike real photometry data where underlying neural dynamics are technically unknown.

Custom MATLAB scripts were used to simulate key components of photometry data and their influence on each other (data available in Ref. 15; simulation code available at https://github.com/philjrdb/RegressionSim). The components of the simulation were in order of operation:

1. A neural dynamic component composed of 100 identical 3 s “excitatory” event transients (waveform taken from Ref. 16), which was tracked by experimental but not isosbestic signals.
2. A photobleaching component, modeled as a double-exponential decay function^5^ that attenuated experimental and isosbestic signals.
3. A movement-related component that stochastically attenuated both experimental and isosbestic signals by a shared percentage.
4. Separate Gaussian noise components added to experimental and isosbestic signals.

The order of these operations was designed to emulate noise, photobleaching, and movement-related transformations of experimental and isosbestic signals across key junctures of the recording set-up (recording site → photodetector → signal processor) [Fig. 3(a)]. Although hemodynamic artifacts were not explicitly included as a component in this simulation, hemodynamic artifacts would be modeled in a similar way to movement (concurrent attenuation of experimental and isosbestic signals). Hence, the movement-related component also serves as a model for hemodynamic artifacts. Neural and photobleaching components were fixed across 10 simulations for a given parameter set (“simulation set”; see the Supplementary Material), whereas movement-related and noise components were stochastic and randomized.

Simulations were used to assess the impact of three key choices made by researchers when obtaining an “artifact-corrected dF/F” from photometry signals: (1) low-pass filtering signals before isosbestic fitting, (2) regression for isosbestic fitting, and (3) using the fitted isosbestic to perform baseline normalization across the session. For each simulation, experimental and isosbestic signals for a 20 min recording session (10 Hz sampling rates) were generated. These signals were then either low-pass filtered (3 Hz) or unfiltered. Isosbestic signals (low-pass filtered or unfiltered) were then fit to corresponding experimental signals using OLS or IRLS [Fig. 3(b)]. IRLS regression was performed by the in-built MATLAB function robustfit, using Tukey’s bisquare as the weighting function. This function is favorable as it entirely ignores large outliers (putative neural dynamics), unlike other weighting functions that only attenuate the influence of large outliers, allowing IRLS to better concentrate the fit onto the artifactual baseline. The narrowness of Tukey’s bisquare is determined by a tuning constant. The default constant is 4.685, but two smaller constants (c = 3 versus 1.4) that make the weighting function narrower (i.e., more aggressive downweighting) were also tested. Larger constants were not tested as this shifts IRLS toward an OLS solution. Finally, fitted isosbestic signals were applied in dF/F [(experimental signal − fitted control) / fitted control] or dF (experimental signal − fitted control) calculations, reflecting the choice to apply versus not apply baseline normalization using the fitted isosbestic signal. In summary, 10 simulations were used to assess the influence of three independent factors: low-pass filter (3 Hz versus none) × baseline correction (dF/F versus dF) × regression (OLS versus IRLS; c=4.685 versus 3 versus 1.4).

The key dependent variable was how much “artifact-corrected” signals (dF/F or dF) reflected the true neural dynamic signal. Given that true and extracted signals are on different scales due to artifact attenuation factors as well as dF/F by definition being a fraction of dF, we normalized true and artifact-corrected signals by dividing whole-session signals by their sum squared deviation from 0 [i.e., they were z-scored relative to 0 (null-Z)^13^]. We assessed average absolute residual (i.e., the difference between normalized true and extracted signals) for event and nonevent (i.e., baseline) periods [Figs. 3(d) and 3(e)]. We also assessed signal accuracy around the first and last events [Figs. 3(f) and 3(g)] to examine how signal processing choices influenced the extraction of signals across the session.

Repeated measures analysis of variance (ANOVA) of residuals across simulations (n=10) revealed that each factor had a significant effect on the accuracy of extracted signals for baseline and event periods. These effects were essentially additive, so we discussed the main effects for each factor in turn.

As expected, applying a 3 Hz low-pass filter to experimental and isosbestic signals resulted in more accurate artifact-corrected signals (Fig. S2 in the Supplementary Material; baseline periods: F(1,9)=12,137.4, p<0.001, $\eta_{G}^{2}=0.99$; event periods: F(1,9)=2,665, p<0.001, $\eta_{G}^{2}=0.97$). Critically, IRLS regression produced more accurate artifact-corrected signals than OLS regression, with this effect scaling with the tuning constant (baseline periods: F(3,27)=37,284.5, p<0.001, $\eta_{G}^{2}=0.99$; event periods: F(3,27)=8,608.8, p<0.001, $\eta_{G}^{2}=0.98$). That is, more aggressive downweighting reliably resulted in better accuracy across parameters [baseline periods: all t(9)>|14.9|, p<0.001; event periods: all t(9)>|10.5|, p<0.001 (Bonferroni-adjusted)]. Finally, baseline normalization via a dF/F calculation outperformed the dF calculation during event periods (F(1,9)=308.4, p<0.001, $\eta_{G}^{2}=0.65$) but performed moderately worse than dF calculation during nonevent periods (F(1,9)=72.80, p<0.001, $\eta_{G}^{2}=0.43$).

The impact of baseline normalization was most pronounced when comparing signal accuracy over the course of the session [Figs. 3(f) and 3(g)]. Baseline normalization via dF/F was substantially better than dF for the first event of the session, regardless of the type of regression used [all t(9)>5.5, p<0.001 (Bonferroni-adjusted)]. dF/F was also superior to dF calculations for the final event of the session, but only significantly so when OLS was used [t(9)=3.1, p<0.05, rest p>0.05 (Bonferroni-adjusted)].

**Fig. 4:**
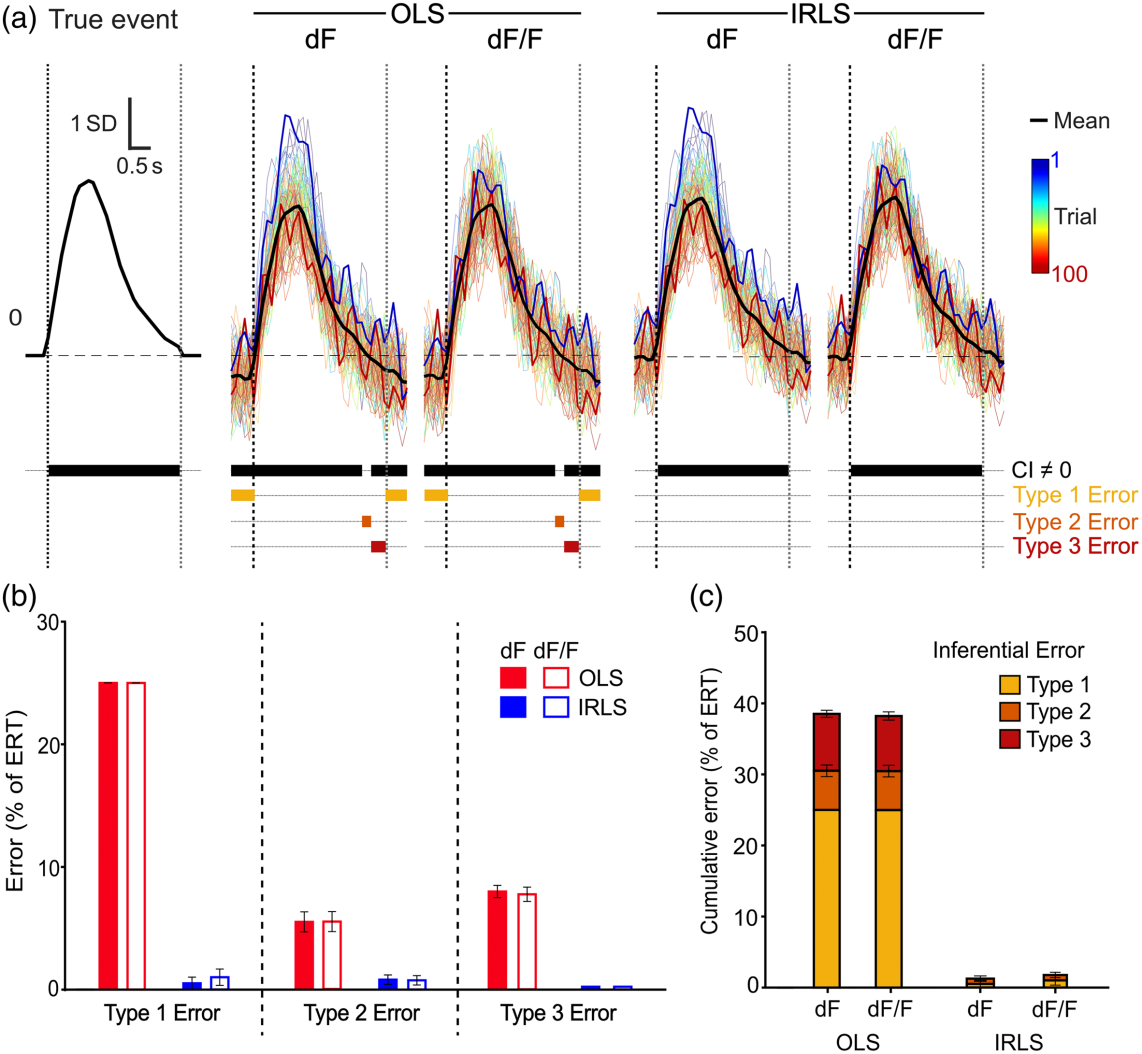
Extracted event-related transients for simulated photometry data (3 Hz low-passed). (a) True versus extracted peri-event signals within a sample simulation. Waveform CI analysis and periods of statistical error displayed beneath each panel. The true 3 s transient (leftmost panel) was constant across the session. OLS-based event transients (dF and dF/F) were downshifted, causing misidentification of peri-event dynamics, whereas IRLS-based transients (dF and dF/F) accurately identified the nature and extent of peri-event dynamics. dF-based transients exhibited a strong spurious change in signals across trials; dF/F-based transients did not. Colored waveforms = signal across trials [first (blue) and last (red) bolded]; black waveform = mean waveform across trials. Vertical dashed lines = start/end of event; horizontal dashed line = session baseline. (b) Mean (±SEM) percentage of waveform in error compared with true transient across 10 simulations. OLS-based transients had more types 1, 2, and 3 inferential errors than IRLS-based transients. (c) Cumulative percentage of mean peri-event waveforms in error across 10 simulations.

Causes for differences in signal accuracy are clear when examining collated true versus extracted peri-event signals [Fig. 4(a)]. The true underlying event transient was a 3 s waveform that was identical in shape and amplitude across the 100 event trials, each flanked with a null baseline of 0. OLS versus IRLS regression, and dF versus dF/F calculations had distinct impacts on the degree to which extracted peri-event signals reflected this.

Baseline normalization had a clear impact on extracted event transients over trials [Fig. 4(a)]. Despite the true transient not changing, there was an obvious trend across trials for dF-based event signals. dF-based transients from initial trials were substantially larger than those from later trials, regardless of whether they were OLS- or IRLS-based. By contrast, baseline normalization via the dF/F calculation mitigated this, restoring the stability of transients across the session.

OLS versus IRLS regression had an independent effect on the accuracy of peri-event signals. Instead of influencing waveforms differentially across trials, regression type affected the average waveform [Fig. 4(a)]. To analyze the significance of this, mean peri-event signals across simulations (n=10) were analyzed using waveform confidence intervals (CIs) [95% t-test CI and 1/3 s temporal threshold]^16^ against the null of 0, as commonly done in the literature.^13^^,^^14^^,^^17^^,^^18^ Analysis of the true peri-event signal confirmed the 3 s waveform period as significantly deviating from 0, whereas pre- and post-event periods (null baseline) did not.

When extracting OLS-based “artifact-corrected” signals around events, mean waveforms (both dF and dF/F) were downshifted relative to the true signal [example from one simulation shown in Fig. 4(a), also evident in Fig. 3(c)], resulting in a misidentification of when and how the peri-event waveform deviated from the null of 0. Specifically, OLS-based analyses failed to identify the full 3 s extent of the positive waveform [type 2 error (false negative)], whereas pre-, late-, and post-event periods were misidentified as being below 0 [type 1 (false positive) and type 3 (opposite inference) errors]. If taken at face value, OLS results would promote misconstrued inferences about peri-event neural dynamics. Alternatively, this reliable “inhibition” prior to the event of interest might be used as an indication of an invalid baseline, which is often combatted by rebaselining each trial to a manually selected pre-event period (critical issues with this discussed below).

By contrast, IRLS-based analyses theoretically bypass these issues by more effectively fitting the isosbestic signal to the artifactual baseline of the experimental signal. Indeed, analysis of IRLS-based transients (both dF and dF/F) accurately identified the 3 s transient from null pre/postperiods without the need for rebaselining or trial-by-trial–based corrections [Fig. 4(a)]. Across simulations, OLS-based transients resulted in more types 1, 2, and 3 errors than IRLS-based transients [Figs. 4(b) and 4(c)].

In summary, these simulations indicate low-pass filtering signals, applying IRLS regression when fitting isosbestic to experimental signals, and using these within a baseline-normalizing dF/F calculation produces better artifact-corrected neural dynamic signals than omitting low-pass filtering, applying OLS regression, and omitting baseline normalization (i.e., only subtracting fitted isosbestic from experimental signals). Although these specific results are demonstrated with a particular set of simulation parameters, their logic applies across photometry scenarios, and we have found these results hold across simulation parameters (simulation set 2: Fig. S3 in the Supplementary Material). We have made a wide range of parameters accessible within the simulation scripts provided at https://github.com/philjrdb/RegressionSim; we welcome further tests.

## 3 Comparing Low-Passed IRLS-Based dF/F against an Alternative Benchmark Pipeline

Although OLS-based dF/F is the default approach within the field,^1^^,^^3^^,^^17^^,^^18^ one analysis pipeline put forward by Martianova et al.^11^ combines smoothing of raw signals, adaptive baseline correction (airPLS), and robust regression to calculate a detrended dF. This pipeline represents a widely used alternative, making it an important benchmark for evaluating our approach. We compared the accuracy of extracted signals produced by this detrended dF with that produced by our recommended signal processing steps (low-passed IRLS-based dF/F).

Briefly, when examining the absolute residuals between extracted and true signals, the results were mixed across simulation sets. Detrended dF generated lower residuals than the low-passed IRLS-based dF/F for most measures within the first simulation set [Fig. S4(a) in the Supplementary Material] but was less accurate across measures for the second simulation set [Fig. S4(e) in the Supplementary Material]. This discrepancy may have arisen from the smoothing algorithm employed by the Martianova et al.^11^ pipeline, which appears to smoothen the raw signals to a greater degree than the 3 Hz low-pass filter [Figs. S4(b) and S4(f) in the Supplementary Material]. Nevertheless, analysis of peri-event waveforms revealed that low-passed IRLS-based dF/F resulted in far fewer inferential errors than the detrended dF across simulation sets [Figs. S4(c)–4(d) and S4(g)–S4(h) in the Supplementary Material].

Overall, our pipeline outperformed the alternative benchmark approach in accurately extracting true signal dynamics. The full details and results of these comparisons can be found in the Supplementary Material.

## 4 Effects of IRLS versus OLS Regression on Real Photometry Data

**Fig. 5:**
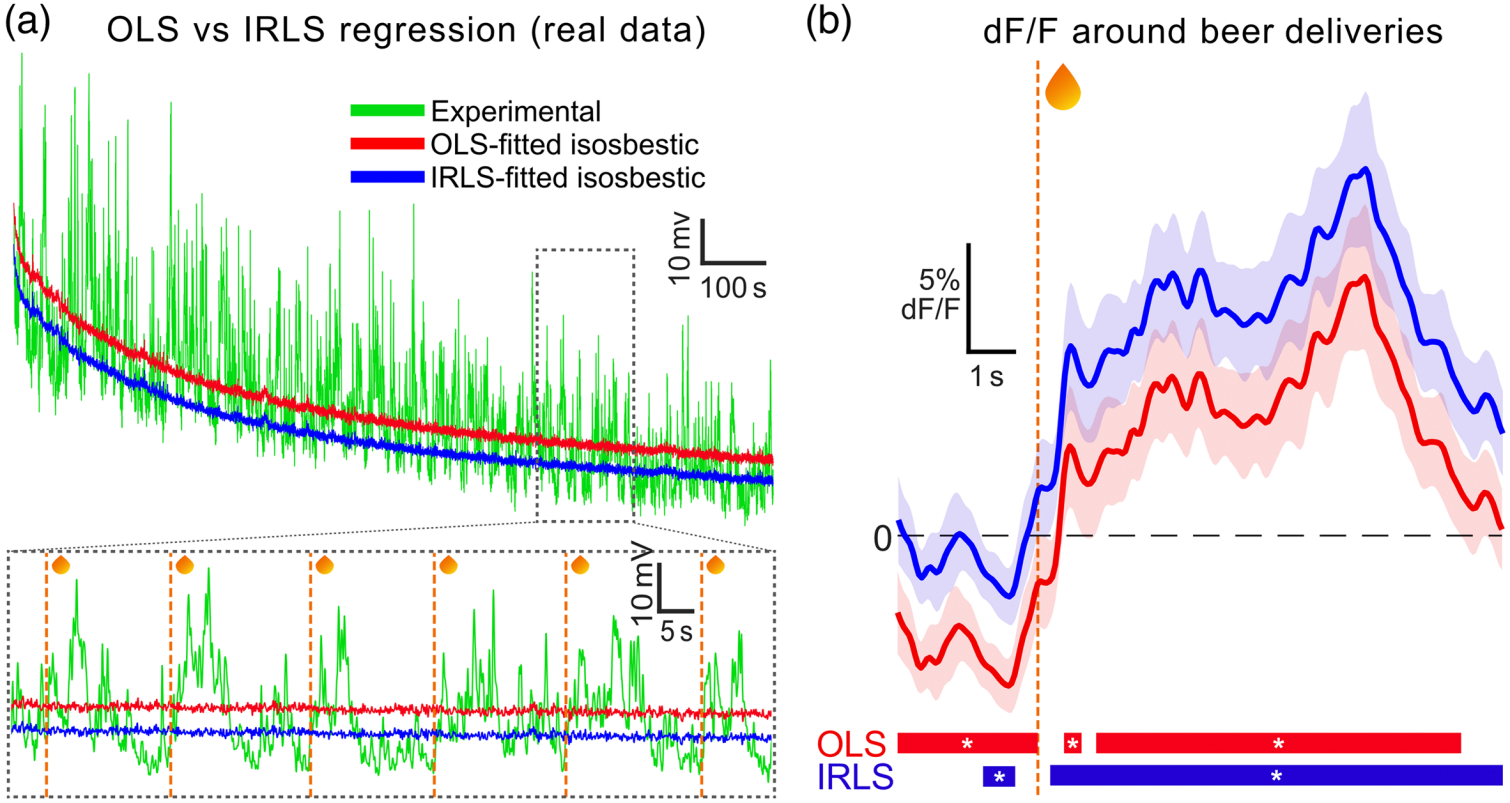
Effect of OLS versus IRLS regression on real photometry data. (a) OLS (red) and IRLS (blue) fits of 405 nm isosbestic signal onto the 465 nm experimental signal (green) across a recording session. Inset: signals from a section of the session (vertical dashed lines = nosepoke-elicited beer delivery times). (b) Mean ± 95% CIs for OLS-based (red) and IRLS-based (blue) dF/F around the onset of beer deliveries (vertical dashed line). Bars at the bottom indicate when dF/F significantly deviates from the session baseline (CI ≠ 0) for more than 1/3 s.

Finally, the relevance of our regression approach for analyses of real fiber photometry data was assessed by comparing the impact of OLS versus IRLS regression on obtained results using real photometry data.^14^ In brief, these data came from an experiment where ${\mathrm{Ca}}^{2+}$ indicator GCaMP6f was selectively expressed in ventral tegmental area (VTA) dopamine neurons of transgenic TH-Cre rats. ${\mathrm{Ca}}^{2+}$-dependent and ${\mathrm{Ca}}^{2+}$-independent GCaMP fluorescence (from 465 and 405 nm excitation light, respectively) was recorded, whereas rats made nosepoke responses for beer. Using exemplar data from a subject during late acquisition phase (data and MATLAB code available at https://github.com/philjrdb/RegressionSim), OLS or IRLS regression [Tukey’s bisquare (c=1.4)] were used to fit the isosbestic signal to the experimental signal (Fig. 5).

OLS and IRLS regressions generated notably different fits of the isosbestic signal onto the experimental signal in this dataset [Fig. 5(a)]. Consistent with conceptual predictions (Fig. 2) and simulations (Fig. 3, Fig. S3 in the Supplementary Material), the IRLS-fitted isosbestic signal more closely followed the base of the experimental signal, whereas the OLS fit was shifted toward the large peaks in the experimental signal that occurred immediately after nosepoke-elicited beer delivery.

This differential fitting impacted the analysis of mean dF/F around beer deliveries [Fig. 5(b)]. Consistent with simulations, OLS-based transients were significantly downshifted relative to IRLS-based transients. For OLS-based dF/F, waveform analysis (95% t-based CIs and 1/3 s temporal threshold^14^) indicated the entire 3 s lead-up to beer delivery as significantly below session baseline, whereas the excitatory component was only reliably detected 1 s after nosepoke-elicited beer delivery. Taken at face value, this might be used to interpret pronounced reductions in VTA dopamine neuron ${\mathrm{Ca}}^{2+}$ prior to nosepokes and delayed excitation to beer delivery. Alternatively, this reliable “inhibition” prior to the event of interest might be used as an indication of an invalid baseline, which could be combatted by rezeroing each trial to a manually selected pre-event period.^1^^,^^17^^,^^18^ However, this choice comes with the problematic assumption that the specified pre-event period acts as a valid baseline across trials. In contrast to this, IRLS-based dF/F around beer delivery exhibited a brief but significant reduction in dF/F immediately before the nosepoke and a robust excitatory transient and thus immediately following beer delivery, without apparent need for rebaselining. Furthermore, this IRLS-based result is more concordant with other findings from the literature.^19^^,^^20^

These findings show that the choice of regression is consequential when analyzing real photometry data. The advantage of IRLS over OLS for this real dataset is speculative as the true ${\mathrm{Ca}}^{2+}$ response of VTA dopamine neurons is technically unknown. However, findings are highly consistent with those from simulated data (Figs. 3 and 4, Fig. S3 in the Supplementary Material) where true parameters were known. This indicates that artifact-corrected signals obtained via IRLS appear to have preferable qualities to those obtained via OLS, with less need for problematic posthoc trial-by-trial adjustments.

## 5 Discussion

Fiber photometry is a powerful neuroscientific measurement technique. However, signals of interest are contaminated by system noise, photobleaching, movement-related, and hemodynamic artifacts that undermine analysis and interpretation. Typically, system noise is attenuated via low-pass filtering, whereas photobleaching, movement-related, and hemodynamic artifacts are addressed using an isosbestic control signal within the early steps of the analysis to obtain an artifact-corrected neural dynamic signal. Despite these solutions being identified in early photometry studies,^3^ approaches for extracting neural dynamics from experimental signals remain relatively unstandardized.^1^ There continues to be substantial variability in whether and how analysis steps are applied across studies and analysis pipelines. Speaking to this, we examined the impacts of key analysis choices—low-pass filtering, baseline normalization (dF/F versus dF), and types of regression (OLS versus IRLS)—on artifact correction. We observed clear and specific advantages of low-pass filtering, IRLS, and dF/F calculations over their alternatives. We discuss these findings and further considerations below.

Low-pass filtering is a ubiquitous, uncontroversial step in photometry analyses. It takes advantage of the fact that biosensors tend to operate on slower timescales than major noise components (biosensors have subsecond but not microsecond kinetics). However, further signal processing, such as the use of bandstop filters, may be warranted if noise components below the ideal low-pass filter threshold are detected.

The common procedure of baseline-normalizing signals using the dF/F calculation stipulated by Lerner et al.^3^ was also shown to be more effective for counteracting photobleaching-related artifacts than simply subtracting the fitted isosbestic from the experimental signal as done in some procedures.^1^^,^^11^^,^^12^ It is important to note that this within-session baseline normalization is separate from and is in fact undermined by detrending and/or normalizing of experimental and isosbestic signals prior to the dF/F calculation.^11^^,^^12^ That is, detrending and/or z-scoring experimental and isosbestic signals preclude their use in dF/F calculations as the original scale, and all positive values of recorded signals are necessary for appropriate baseline normalization. However, once dF/F is calculated, subsequent detrending and normalization steps for the artifact-corrected dF/F are possible (discussed below).

A key argument we put forward here is that standard OLS regression is not ideal for fitting the isosbestic signal to the experimental signal. This is because it overfits the isosbestic signal to the experimental signal’s neural dynamic component while underfitting to the artifactual component it is intended to track. We show that robust regression, specifically IRLS regression, is conceptually and evidently better suited for this purpose. Interestingly, we found that lower tuning constants (i.e., more aggressive downweighting) produced better results across the constants tested. It is unclear whether this trend holds true for even lower tuning constants or the degree to which this trend applies across scenarios. However, our findings suggest that stronger over weaker applications of IRLS can be beneficial for extracting neural dynamics from experimental signals.

In addition to extracting a more accurate neural dynamic signal, IRLS also tracks the baseline of the neural dynamic signal more closely. This reduces the need for problematic trial-by-trial rebaselining. This arbitrary extra step requires the assumption that the designated pre-event period serves as a valid baseline (i.e., is free of neural dynamics that might systematically or unsystematically skew trial data, including potential pre-event inhibitory dynamics). This assumption can be especially problematic for tasks in which animal behavior and potential anticipatory neural dynamics pervade the recording session (e.g., free operant tasks^13^). IRLS presents an effective way to bypass these issues.

However, local rebaselining may still be required when longer term fluctuations in neural dynamics skew the analysis of phasic signals. One option is to detrend these fluctuations after IRLS-based dF/F extraction. In this case, we recommend employing a smoothed moving median over a moving mean as the latter will overfit the moving average to neural dynamics (as per OLS). Relatedly, if further normalization of the artifact-corrected dF/F is desired, we recommend using the null-Z method (as employed in this study) instead of standard z-scoring to normalize dF/F. This is because standard z-scoring reverts the signal mean to 0 (as per OLS), which may not reflect the IRLS-derived baseline. The null-Z avoids this, normalizing the signal relative to the IRLS-derived baseline.

Finally, a consideration not previously mentioned is the common step of excluding invalid recording periods, such as nonrecording or patchcord disconnection periods. This logical step should be done as the first preprocessing step as these invalid periods and transitions between valid and invalid recording periods can adversely affect signal processing and isosbestic regressions steps.

## 6 Conclusion

Taken together, we recommend measuring an isosbestic control signal within the same subject [either via the isosbestic wavelength of the biosensor or a spectrally distinct static fluorophore (e.g., tdTomato)] and applying four analysis steps to better extract the true neural dynamic component of experimental biosensor signals:

1. Exclude invalid recording periods (e.g., logged disconnections) from further analysis.
2. Apply a low-pass filter to remove high-frequency noise components from experimental and isosbestic signals. The cut-off for this filter will depend on the kinetics of the biosensor and problematic noise components.
3. Fit the isosbestic signal to the experimental signal using IRLS regression. We recommend using Tukey’s bisquare with a low-tuning constant.
4. Calculate a baseline-normalized dF/F using the equation outlined by Lerner et al.^3^

These initial analysis steps are not exhaustive. Additional processing steps may be needed, as discussed above. However, these four specific steps are beneficial over existing alternatives and should apply to most use cases. Therefore, we argue that these steps could serve as a standard component across photometry analysis pipelines.

## Supplementary Material

10.1117/1.NPh.12.2.025003.s01

## Data Availability

All data and code used in this article are made available at https://osf.io/z6c5b/ and https://github.com/philjrdb/RegressionSim.
