# Supplementary material for: Obtaining artifact-corrected signals in fiber photometry via isosbestic signals, robust regression, and dF/F calculations
Source: Neurophotonics. 2025 Mar 31;12(2):025003. doi: 10.1117/1.NPh.12.2.025003 (PMC11957252; doi:10.1117/1.NPh.12.2.025003)
Supplement: Supplementary file 1 [file NPh_012_025003_SD001.pdf]

Supplementary Materials

1 Supplementary Data: Demonstration of light loss during fiber bending

It is well known that the transmission efficiency of light through a fiber optic cable is affected by fiber bending[6,7]. Given typical fiber photometry experiments use a flexible fiber optic patchcord to deliver/collect light while animals behave, movement-related bending in the patch cable can introduce artifactual signal change (**Fig. 1**). To visually demonstrate this phenomenon, we captured images of a fiber cable (0.39NA Ø400µm; [FT400EMT, ThorLabs]) delivering 465nm light while it was straight versus bending (**Fig. 1B, S1A**). Photographs were taken of the same middle section of the cable from the same position with identical image acquisition settings (Canon EOS 6D, 1/50sec, f/4, ISO-1000); the only modified parameter was whether this portion of the cable was straight or bending (~90° over ~20cm). There were no other light sources, and the image was taken perpendicular to the cable (the direction of light transmission), so any light captured by the camera would be of light escaping from that portion of cable (instead of continuing down the cable as generally intended).

We observed that the bending portions of the cable were visibly brighter than straight portions (**Fig. 1B, S1A**). To enhance visualization, we also provide these images with adjusted color grading (converted to 8-bit grayscale and re-colored via look-up table [LUT]) to highlight differences in brightness intensity (**Fig. S1B**). So, more light appears to be escaping from bending portions of the cable than straight portions.

To quantify the relationship between fiber curvature and light loss, we performed an image analysis using ImageJ[22]. Segmented lines were drawn along the midline of each fiber and fitted with a spline (**Fig. S1C**). A regional intensity analysis was then conducted by averaging pixel

brightness across 3mm segments along the fiber's length. To quantify fiber curvature, we used the Law of Cosines to compute the absolute change in angle between each 3mm segment. The relationship between brightness and curvature is plotted in **Fig. S1D-E**, showing that variations in curvature correspond closely with changes in brightness. We show that there is a strong relationship between bending and brightness across images (linear fit calculated with RStudio, v4.3.2).

In summary, fiber bending causes observable changes in light transmission efficiency by allowing more light to escape from fiber optic cables. Given fiber bending is a dynamic, continuous, and largely unavoidable feature of fiber photometry recordings, a procedure for tracking this source of artifacts is critical.

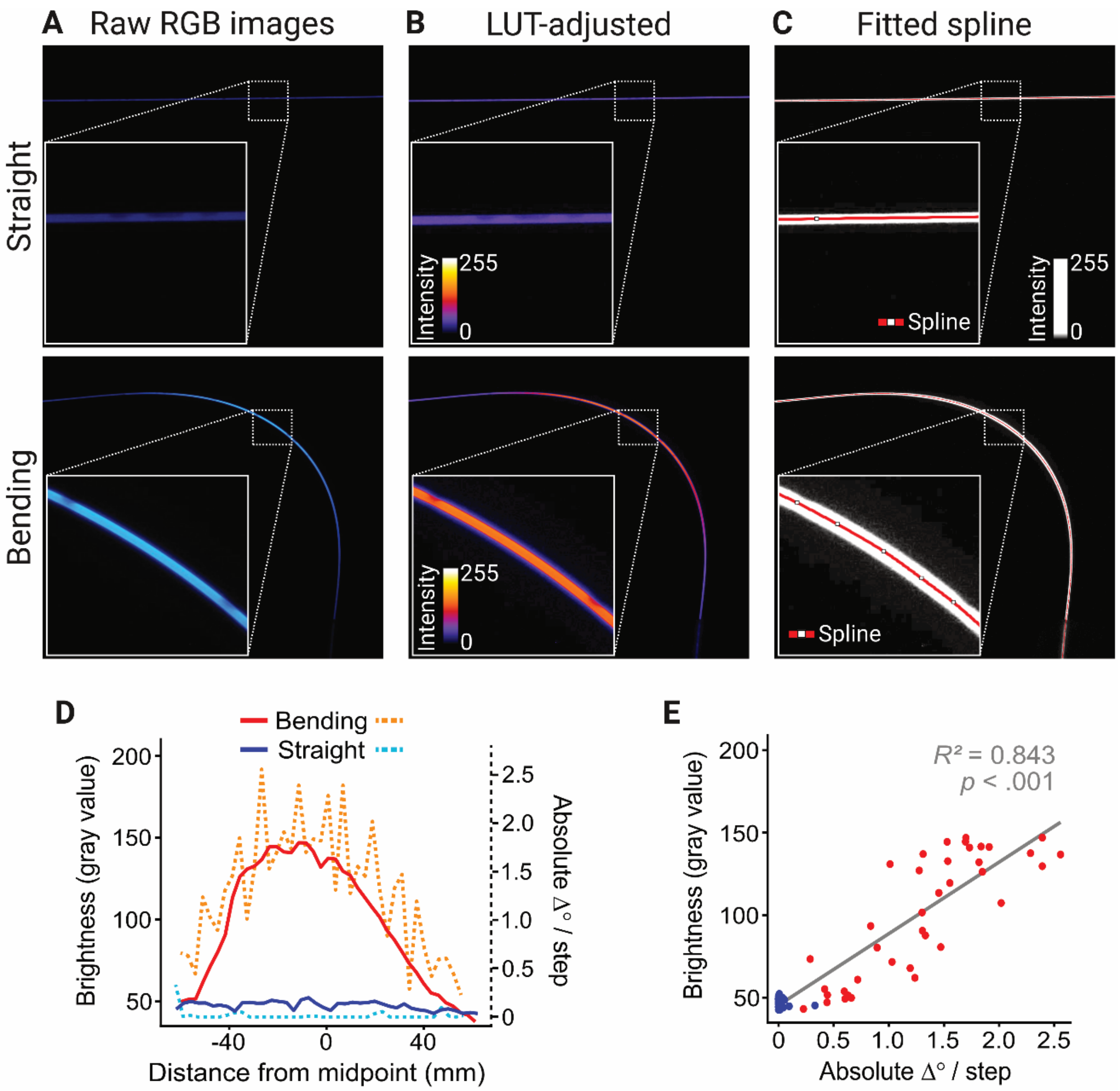

Figure S1. Relationship between fiber bending and light loss. **[A]** Raw images of straight (*top*) and bending (*bottom*) optic fibers (as per Fig. 1B). Very little light is observable (i.e., is escaping) from straight portions of the fiber; notably more light is observable in bending portions. **[B]** The same images but re-colored via look-up table (LUT). **[C]** A spline was fit along the center of the fiber per image to allow quantification of brightness and curvature along the fiber. **[D]** Brightness (average gray value; *solid lines*) and curvature (absolute ° change; *dotted lines*) across 3mm segments of bending (*red lines*) and straight (*blue lines*) fibers. Brightness (i.e., escaping light) corresponded strongly with degree of bending in each segment of fiber. **[E]** There was a strong positive relationship (grey line = linear fit) between brightness and fiber curvature across bending (*red dots*) and straight (*blue dots*) fibers.

2 Supplementary Methods: Simulation of fiber photometry data

Artificial fiber photometry data was generated using custom MATLAB scripts (code available at https://github.com/philjrdb/RegressionSim). These scripts offer a sandbox environment to simulate fiber photometry data with a range of parameter options. To assess the robustness of our findings, we conducted two separate sets of 10 simulations. One set of 10 simulations were run to produce the data shown in **Fig. 3-4**, **Fig. S2** and **Fig. S4 (A-D)**. A second set of 10 simulations were run to produce the data shown in **Fig. S3** and **Fig. S4 (E-H).**

Across simulation sets, experimental and isosbestic signals were simulated as recordings from a 20min session (10 Hz sampling rate). Signals were constructed via four components:

1. Neural dynamic component

The neural dynamic component of the experimental signal (saved as "neural_component") was modelled as a vector of zeros, to which 100 identical event-related transients (ERT) were added. ERT onsets were distributed evenly between 10sec and 1100sec. The isosbestic signal was modelled as a vector of zeros with no ERTs added.

Each ERT was based on an exemplar waveform vector used in[15]. Two parameters were used to modify the size of each ERT: an "ERT_peak_multiplier" parameter that adjusted the ERT waveform in 1 dimension (scaled the height of ERTs), and an "ERT_coeff" parameter that adjusted the waveform in 2 dimensions (scaled the height and length of ERTs). For the first set of simulations, ERT_peak_multiplier = 20 and ERT_coeff = 3. For the second set of simulations, ERT_peak_multiplier = 8 and ERT_coeff = 8.

2. Photobleaching component

The photobleaching component for experimental and isosbestic signals (saved as "exp_decay_component" and "iso_decay_component", respectively) were modelled as a double-exponential decay function[5] which started at 1 and decayed across the session towards a specified % attenuation parameter ("exp_decay_base", "iso_decay_base"). The decay function was determined by 2 rate parameters per signal ("exp_decay_rate1", "exp_decay_rate2", "iso_decay_rate1", "iso_decay_rate2") via the following equation: $([1 - \text{rate1}]^t + [1 - \text{rate2}]^t) / 2$, with *t* representing session time.

For simulation set 1, exp_decay_rate1 = 0.02, exp_decay_rate2 = 0.002, exp_decay_base = 40, iso_decay_rate1 = 0.02, iso_decay_rate2 = 0.002, and iso_decay_base = 40. For simulation set 2, exp_decay_rate1 = 0.02, exp_decay_rate2 = 0.002, exp_decay_base = 30, iso_decay_rate1 = 0.02, iso_decay_rate2 = 0.002, and iso_decay_base = 40.

3. Movement-related component

Movement artifact component (saved as "movement_component") was modelled as a vector of random numbers (generated via *rand* function) which was then smoothed using a 0.1 Hz low-pass filter (*lowpass* function) and scaled to fluctuate between 1 and specified maximum % attenuation ("movement_attenuation" parameter). This component was shared between the experimental and isosbestic signals in a simulation, as would be the case with real fiber photometry recordings, but was randomized across simulations. Across simulation sets, movement_attenuation = 50.

4. Noise component

Noise artifact components per signal (saved as "exp_noise_component" and "iso_noise_component") were modelled as a vector of normally distributed random numbers (generated via *randn* function), which was then multiplied by a specified scaling parameter ("noise_factor"). Across simulation sets, noise_factor = 2.

In addition to these components, a parameter representing tonic/background fluorescence (i.e., raw signal baseline) was included per signal ("exp_base", "iso_base"). For the first set of simulations, exp_base = 200 and iso_base = 80. For the second set of simulations, exp_base = 300 and iso_base = 120.

The four components were combined with signal baselines to generate "recorded" experimental and isosbestic signals as follows:

Experimental signal = ((neural_component + exp_base) x exp_decay_component) x movement_component + exp_noise_component

Isosbestic signal = (iso_base x iso_decay_component) x movement_component + iso_noise_component

3 Supplementary Methods: Processing and analyses of simulated signals

To examine the influence of low-pass filtering signals before analysis, 3 Hz low-passed versions of experimental and isosbestic signals were obtained using MATLAB's in-built *lowpass* function (infinite impulse response filter, transition band steepness = 0.95).

A fitted isosbestic signal was obtained by regressing the isosbestic signal onto the raw experimental signal using in-built *polyfit* and *polyval* functions for OLS, and *robustfit* and *polyval* functions for IRLS. In the case of IRLS regression, Tukey's bisquare was used as the weighting function and separate regressions were run using tuning constants (c) of 1.4, 3 and 4.685.

Fitted isosbestic signals (OLS- or IRLS-based) were used to obtain "artifact-corrected" dF and dF/F signals using the following equations:

dF = (experimental signal - fitted control)

dF/F = (experimental signal - fitted control)/fitted control

dF, dF/F and true (neural_component) signals were each normalized by dividing them by their sum squared deviation from 0 (custom *nullZ* function).

The average absolute residual (mean absolute difference) between normalized true and "artifact-corrected" signals were calculated for event and non-event (baseline) periods. They were also calculated for the first and last event periods of the session individually. Statistical analyses on the absolute residuals were conducted in RStudio (version 4.3.2) using the *ez* package[23]. (2) x (2) x (4) repeated measures ANOVA was conducted to assess the effects of low-pass filtering (low-pass vs no low-pass), baseline normalization (dF vs dF/F) and regression type (OLS vs IRLS [c = 1.4 vs 3 vs 4.685]) on residuals for event and baseline periods across simulations. Effect sizes were reported via generalized eta squared (η_G^2), which provides an unbiased estimate of proportion

of variance explained by a factor in multifactorial repeated measure designs. Where necessary, Bonferroni-adjusted pairwise t-tests were used to make pairwise comparisons.

Mean peri-event signals across simulations were analyzed using waveform confidence intervals (95% t-test CI, 1/3sec temporal threshold[15]) and inferential errors were identified by comparing the results of these confidence intervals to the true signal. Type 1 errors were defined as time points where the peri-event waveform was flagged as significantly different from 0 despite the true neural signal being 0. Type 2 errors were defined as time points where the peri-event waveform was not flagged as significantly different from 0, despite the true neural signal not being 0. Finally, Type 3 errors were defined as time points where the peri-event waveform was flagged as significantly different from 0 but in the opposite direction to the true neural signal (e.g., peri-event waveform flagged as <0 when true neural signal >0).

4 Supplementary Results: Effects of low-pass filtering (simulation set 1)

We assessed whether a low-pass filter improved the extraction of neural dynamic signal. As expected, applying a 3Hz low-pass filter to experimental and isosbestic signals reliably improved artifact-corrected signal accuracy relative to not low-passing signals (baseline periods: $F(1,9) = 12{,}137.4$, $p<.001$, $\eta_G^2 = 0.99$; event periods: $F(1,9) = 2{,}665$, $p<.001$, $\eta_G^2 = 0.97$). This was true across all combinations of OLS vs. IRLS and dF vs dF/F; effect of low-pass filtering for OLS and IRLS ($c = 1.4$) dF/F are depicted in **Fig. S2** (OLS baseline: $t(9) > 70.9$, $p<.001$; IRLS baseline: $t(9) > 60.4$, $p<.001$; OLS overall event: $t(9) > 48.8$, $p<.001$; IRLS overall event: $t(9) > 40.5$, $p<.001$), OLS first event: $t(9) > 2.4$, $p<.05$; IRLS first event: $t(9) > 6.2$, $p<.01$; OLS last event: $t(9) > 8.3$, $p<.001$; IRLS last event: $t(9) > 7.0$, $p<.001$).

Low-pass filtering had little effect on the general shape and position of the mean peri-event waveform relative to baseline (example simulation shown in **Fig. S2B**). Nevertheless, it did smooth individual trials, resulting in a smoother mean ERT and less spurious variance per timepoint. This is unsurprising, given the function of a low-pass filter is to suppress high-frequency fluctuations, which smooths signals and reduces variance driven by high-frequency noise. This reduced variance allowed more timepoints that deviated from the null of 0 to be detected as significant (**Fig S2C-D**). The impact of this was double-edged, depending on regression type. It reduced Type 2 errors (failure to detect a true ERT) for both OLS- and IRLS-based dF/F. However, it increased the incidence of Type 3 errors (detection of ERT in opposite direction to true ERT) for OLS-based dF/F due to the OLS-based waveform being downshifted relative to the true ERT.

In summary, applying a low-pass filter produced a less noisy signal that better reflected the true underlying neural dynamic signal. This improved the power of waveform analyses, which

reduced Type 2 error generally, but increased Type 3 error in OLS-based analyses that were vulnerable to this type of error.

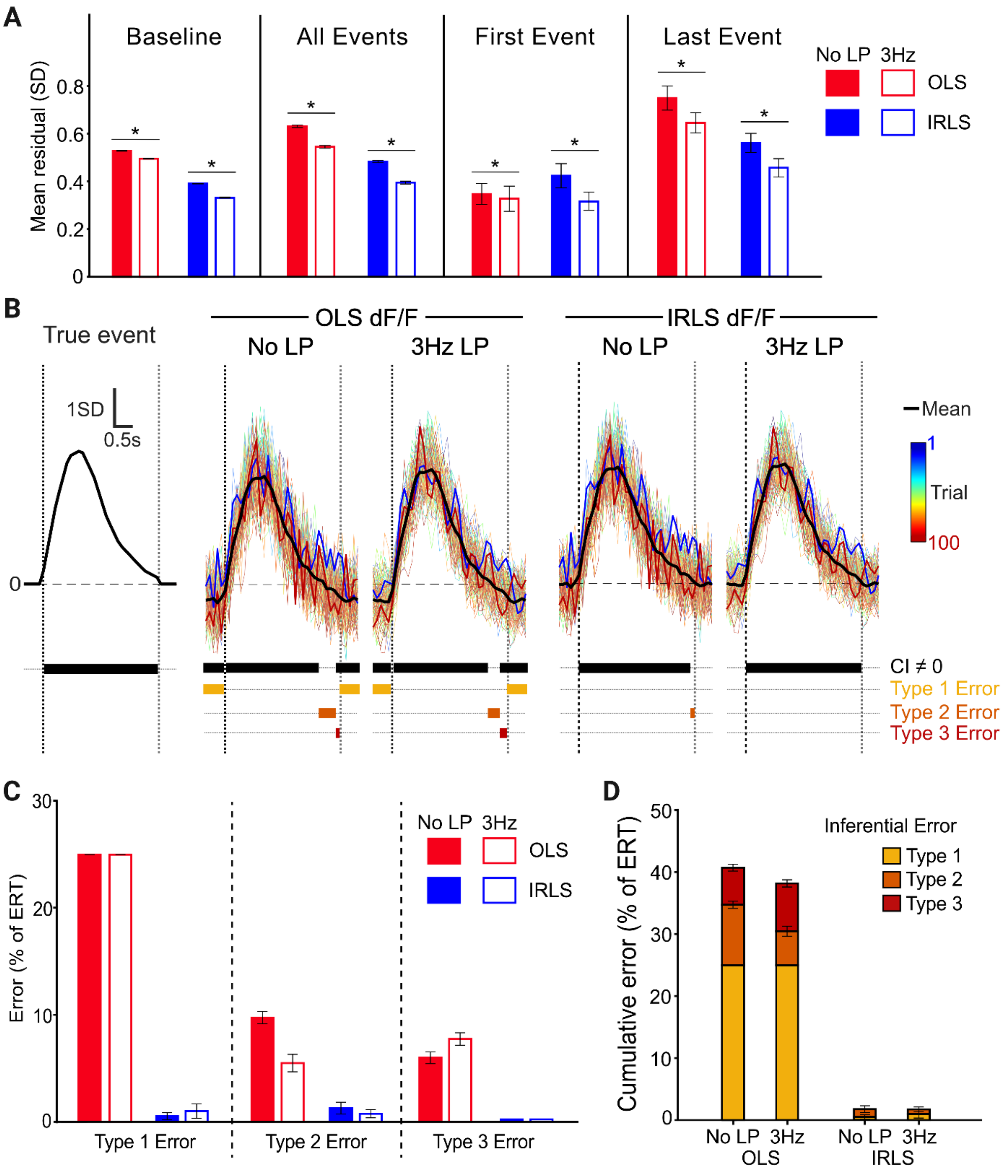

Figure S2. Comparing the effect of low-pass filtering on simulated fibre photometry data. **[A]** Mean (±95% CI) absolute residuals of low-passed (3 Hz) versus non-low-passed OLS- and IRLS-based dF/F signals during non-event

periods (first panel), all events periods (second panel), first event (third panel), and last event (last panel), across 10 simulations. Applying a 3 Hz low-pass filter to signals reliably improved accuracy. *p < .05. **[B]** True vs. extracted peri-event signals for a sample simulation (OLS- and IRLS-based dF/F). Colored waveforms = signal across trials (first [blue] and last [red] bolded); black waveform = mean waveform across trials. Vertical dashed lines = start/end of event; horizontal dashed line = session baseline. The true 3sec event-related transient (ERT; leftmost figure) was constant across the session. Mean OLS-based ERT was downshifted relative to the true and IRLS-based ERT (see also Figure 4). Low-pass filtering had little effect on this pattern, but did result in a smoother signal that more closely resembled the true ERT. By reducing artifactual high-frequency fluctuations, low-pass filtering reduced variance, causing signals across trials to be more tightly clustered around the mean ERT. **[C]** Mean (±SEM) percentage of mean peri-event waveforms in error compared to true transient across 10 simulations. By reducing ERT variance, low-pass filtering reduced the incidence of Type 2 errors for both OLS and IRLS-based dF/F, but also increased the incidence of Type 3 errors for OLS-based analyses. **[D]** Cumulative percentage of mean peri-event waveforms in error across 10 simulations.

5 Supplementary Results: Simulations with different parameters (simulation set 2)

To assess the generalizability of the results from the first set of simulations, we ran a second set of simulations with different parameters (**Fig. S3**), based on a data from a different photometry study[13]. Compared to the first set of simulations, the second set had higher baseline fluorescence for the experimental and isosbestic signals, resulting in greater absolute photobleaching decay, and ERTs were larger and longer in duration

The different parameters used in the 2nd set of simulations produced different results (**Fig. S3**) to the 1st simulation set (**Figs. 3–4**), but the overarching conclusions remain consistent. Although not shown in Fig. S2 for sake of brevity, applying a 3Hz low-pass filter to experimental and isosbestic signals resulted in more accurate artifact-corrected signals (baseline periods: $F(1,9) = 199.3$, $p<.001$, $\eta_G^2 = 0.10$; event periods: $F(1,9) = 10{,}360$, $p<.001$, $\eta_G^2 = 0.88$), recapitulating

findings described in the previous section (**Fig. S2**). As per the 1st simulation set, IRLS regression yielded substantially more accurate artifact-corrected signals than OLS regression, with this effect scaling with the IRLS tuning constant (**Fig. S3C-F**; baseline: $F(3,27) = 12{,}184$, $p<.001$, $\eta_G^2 = 0.99$; all events: $F(3,27) = 11{,}897$, $p<.001$, $\eta_G^2 = 0.99$; first event: $F(3,27) = 334.24$, $p<.001$, $\eta_G^2 = 0.87$; last event: $F(3,27) = 236.53$, $p<.001$, $\eta_G^2 = 0.72$). Baseline normalization via dF/F calculation had minor effects on the accuracy of extracted signals. As per the 1st simulation set, dF performed slightly better than dF/F for baseline periods (**Fig. S3C**; $F(1,9) = 193.99$, $p<.001$, $\eta_G^2 = 0.06$), but the reverse was true for event periods (**Fig. S3D**; $F(1,9) = 31.83$, $p<.001$, $\eta_G^2 = 0.11$). Unlike the 1st simulation set, baseline normalization had no significant effect on signal accuracy around first (**Fig. S3E**; $F(1,9) = 1.24$, $p=.29$, $\eta_G^2 = 0.02$) or last events (**Fig. S3F**; $F(1,9) = 0.03$, $p=.85$, $\eta_G^2 < 0.01$) for the 2nd simulation set.

Pre-processing choices had similar effects on event-related transient analysis for the 2nd simulation set (**Fig. S3G-I**) as the 1st simulation set (**Fig. 4**). As per the 1st simulation set, OLS-based event waveforms were downshifted relative to the true peri-event signal (**Fig. S3G**). This resulted in a broad misidentification of when and how the peri-event waveform deviated from the null of 0 (i.e., Type 1, 2, and 3 errors) (**Fig. S3H-I**). By contrast, IRLS-based waveforms were more accurately positioned, with analyses more closely reflecting the true peri-event signal. Cumulatively, OLS-based transients resulted in far more inferential errors than IRLS-based transients, particularly Type 3 errors (**Fig. S3H-I**). As per the 1st simulation set, baseline normalization via dF/F calculation had little effect on inferential errors in the analysis of mean peri-event waveforms. However, dF/F calculations did mitigate the spurious change in event-related signals across trials (**Fig. S3G**), although this effect was more subtle in the 2nd simulation set compared to the 1st (**Fig. 4A**).

In summary, the markedly different parameters used for the 2nd simulation set produced distinct results from the 1st simulation, but the key findings were recapitulated. Low-pass filtering signals, using IRLS regression to fit isosbestic to experimental signals, and using these within a baseline-normalizing dF/F calculation, produced better artifact-corrected signals than their alternatives.

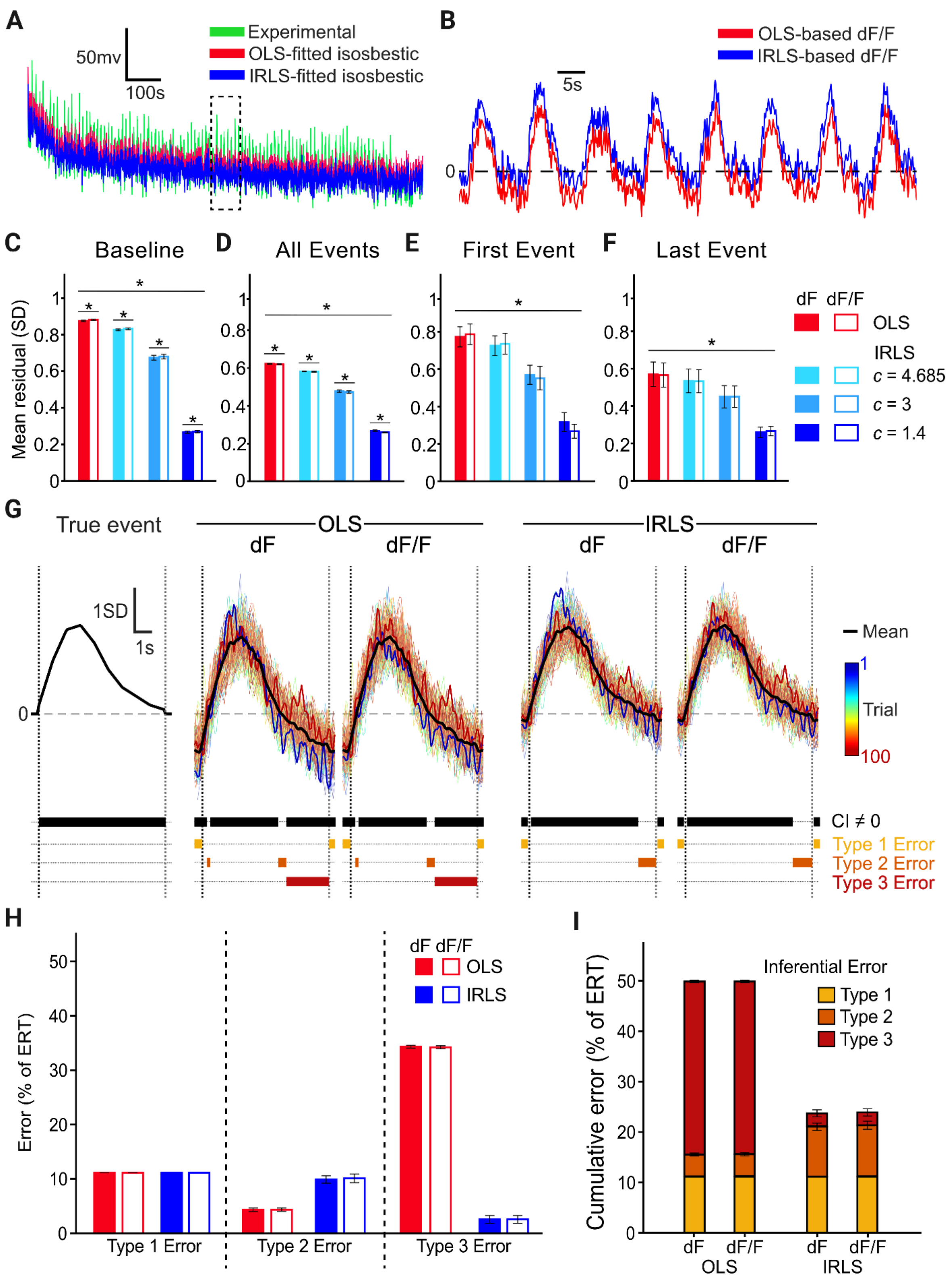

Figure S3. Results of simulations with different underlying signal parameters (simulation set 2 [3Hz low-passed]).

[A-B] Example fitting of simulated isosbestic onto corresponding experimental signal using OLS versus IRLS

regression (whole-session **[A]**, and section indicated by dashed box **[B]**). A modest but consequential difference in baseline is observed. **[C-F]** Mean (±95% CI) absolute residuals during **[C]** non-event baseline, **[D]** across events, **[E]** first event, and **[F]** last event, following OLS vs. IRLS (*c* = tuning constant) regressions and dF vs. dF/F calculations. $*p < .05$. **[G]** True vs. extracted peri-event signals within a sample simulation. Colored waveforms = signal across trials (first [blue] and last [red] bolded); black waveform = mean waveform across trials. Vertical dashed lines = start/end of event; horizontal dashed line = session baseline. Waveform confidence interval (CI) analysis and periods of statistical error displayed beneath each panel. The true 8sec transient (leftmost panel) was constant across the session. OLS-based transients were downshifted, causing more misidentification of peri-event dynamics than IRLS-based transients. More change in signal across trials (spurious) is observed for dF-based compared to dF/F-based transients. **[H]** Mean (±SEM) percentage of waveform in error compared to true transient across 10 simulations. **[I]** Cumulative percentage of mean peri-event waveforms in error across 10 simulations. OLS-based transients had more inferential errors than IRLS-based transients.

6 Supplementary Results: Comparing low-passed IRLS-based dF/F against an alternative benchmark procedure

We compared how the processing steps recommended here (low-passed IRLS-based dF/F) performed against an alternative pipeline put forward by Martianova et al.[11]. Their pipeline involves: 1) smoothing raw signals using a moving mean, 2) baseline correction via the adaptive iteratively reweighted Penalized Least Squares algorithm (https://github.com/zmzhang/airPLS), 3) z-scoring the baseline-corrected signals, 4) fitting the z-scored isosbestic signal to the z-scored experimental using non-negative robust linear regression, 5) calculating a dF score.

We obtained MATLAB scripts from Martianova et al.[11] (original scripts available at: https://github.com/katemartian/Photometry_data_processing, adapted versions available at: https://github.com/philjrdb/RegressionSim), applied them within our simulations to obtain a detrended dF, and compared this against our recommended low-passed IRLS-based dF/F. For the detrended dF, we used a smoothing window (smooth_win) of 3 to correspond to our 3Hz low-pass filter. For the airPLS function, we used the default parameters: lambda = 5e9, order = 2, wep = 0.1, p = 0.5, itermax = 50. Corresponding with the analyses carried out in the main text, we normalized the detrended dF relative to zero (nullZ) and examined how well the artifact-corrected signal resembled the true underlying neural dynamic across both simulations sets (**Fig. S4**).

Accuracy of artifact-corrected (detrended dF vs. low-passed IRLS-based dF/F) signals, as measured through absolute residual, produced mixed results across simulation sets. For the 1st simulation set, the detrended dF was more accurate than the IRLS-based dF/F during baseline periods ($t(9) = 42.09$, $p<.001$), event periods overall ($t(9) = 29.52$, $p<.001$), and the last event of the session ($t(9) = 13.93$, $p<.001$), but much less accurate for the first event of the session ($t(9) = 4.41$, $p<.05$) (**Fig. S4A**). For the 2nd simulation set, IRLS-based dF/F was much more accurate

than detrended dF across each period type (**Fig. S4E**; baseline: $t(9) = 204.9$, $p<.001$; all events: $t(9) = 404.5$, $p<.001$; first event: $t(9) = 11.7$, $p<.001$; last event: $t(9) = 8.8$, $p<.001$).

When examining the accuracy of peri-event waveform analyses, detrended dF waveforms were downshifted relative to the true signals (subtly for 1st simulation set [**Fig. S4B**], noticeably for 2nd set [**Fig. S4F**]), as found with OLS-based waveforms (**Fig. 4**). This resulted in greater misidentification of when and how the peri-event waveform deviated from the null of 0, compared to IRLS-based waveforms (**Fig. S4C-D**, **G-H**).

Taken together, these results indicate low-passed IRLS-based dF/F is, in sum, preferable to detrended dF (as outlined by Martianova et al.[11]) in estimating true underlying signal dynamics. This shows the suggested steps of low-pass filtering signals, applying IRLS regression, and using signals within a baseline-normalizing dF/F calculation, serve as an improvement over existing popular workflow.

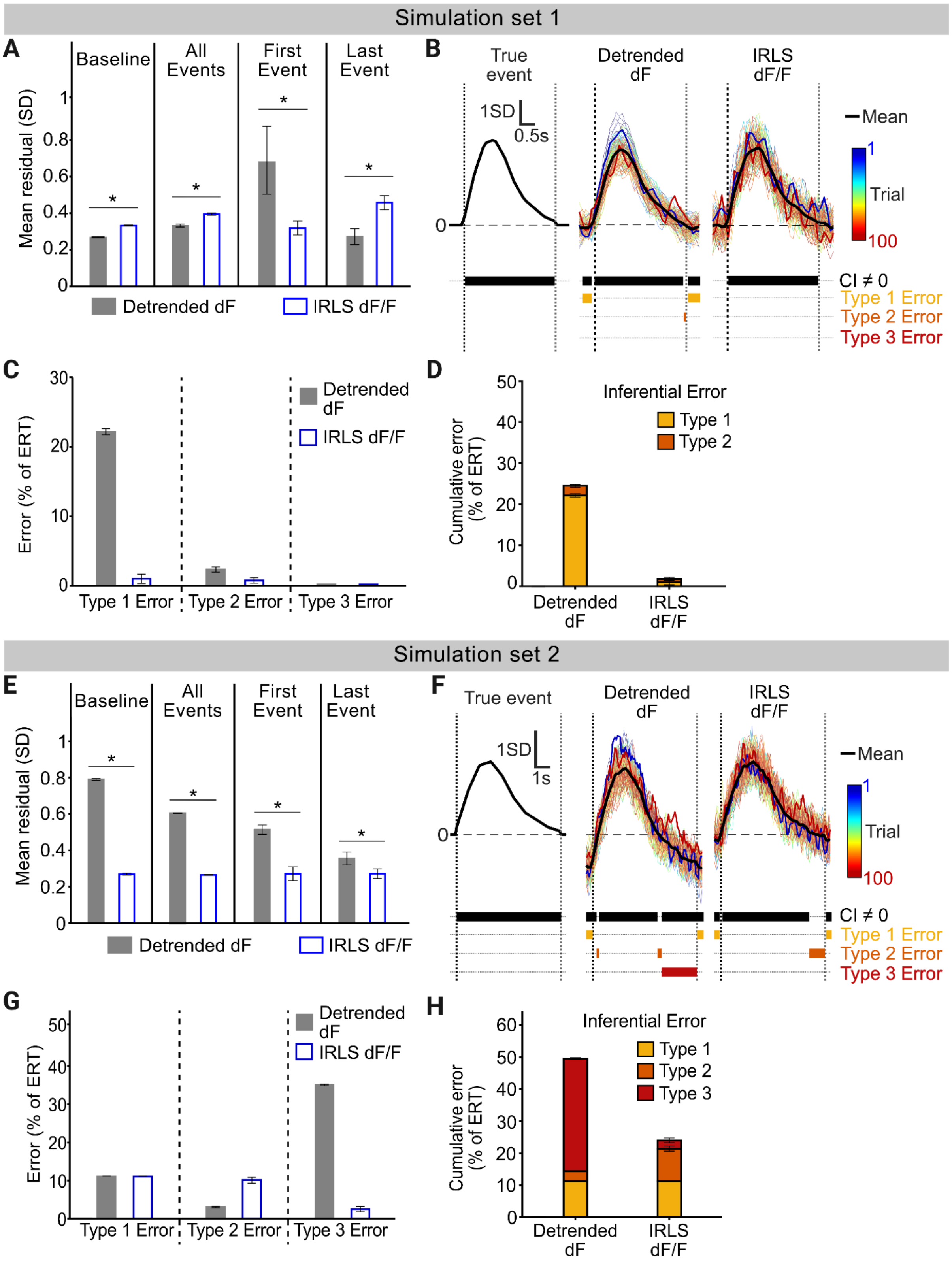

Figure S4. Comparison of recommended low-passed IRLS-based dF/F signals vs. detrended dF signals obtained via an established alternative analysis pipeline[11] for 1st (**[A-D]**) and 2nd (**[E-H]**) simulation sets. **[A,E]** Mean (±95% CI)

absolute residuals for artifact-corrected signals during non-event (baseline; first panel), all events (second panel), first event (third panel), and last event (last panel) periods across 10 simulations. **p* < .05. **[B,F]** True vs. extracted peri-event signals within a sample simulation. Colored waveforms = signal across trials (first [blue] and last [red] bolded); black waveform = mean waveform across trials. Vertical dashed lines = start/end of event; horizontal dashed line = session baseline. Waveform confidence interval (CI) analysis and periods of statistical error displayed beneath each panel. Across simulation sets, detrended dF transients (middle panel) were downshifted relative to the true (left panel) and IRLS dF/F transients (right panel). A spurious change in signals across trials was more evident for detrended dF than IRLS dF/F. **[C,G]** Mean (±SEM) percentage of waveform in error compared to true transient across 10 simulations. **[D,H]** Cumulative percentage of mean peri-event waveforms in error across 10 simulations. Detrended dF had more inferential errors than IRLS dF/F transients.

Caption List

Figure S1. Relationship between fiber bending and light loss. **[A]** Raw images of straight (*top*) and bending (*bottom*) optic fibers (as per Fig. 1B). Very little light is observable (i.e., is escaping) from straight portions of the fiber; notably more light is observable in bending portions. **[B]** The same images but re-colored via look-up table (LUT). **[C]** A spline was fit along the center of the fiber per image to allow quantification of brightness and curvature along the fiber. **[D]** Brightness (average gray value; *solid lines*) and curvature (absolute ° change; *dotted lines*) across 3mm segments of bending (*red lines*) and straight (*blue lines*) fibers. Brightness (i.e., escaping light) corresponded strongly with degree of bending in each segment of fiber. **[E]** There was a strong positive relationship (grey line = linear fit) between brightness and fiber curvature across bending (*red dots*) and straight (*blue dots*) fibers.

Figure S2. Comparing the effect of low-pass filtering on simulated fibre photometry data. **[A]** Mean (±95% CI) absolute residuals of low-passed (3 Hz) versus non-low-passed OLS- and IRLS-based dF/F signals during non-event periods (first panel), all events periods (second panel), first event (third panel), and last event (last panel), across 10 simulations. Applying a 3 Hz low-pass filter to signals reliably improved accuracy. *$p < .05$. **[B]** True vs. extracted peri-event signals for a sample simulation (OLS- and IRLS-based dF/F). Colored waveforms = signal across trials (first [blue] and last [red] bolded); black waveform = mean waveform across trials. Vertical dashed lines = start/end of event; horizontal dashed line = session baseline. The true 3sec event-related transient (ERT; leftmost figure) was constant across the session. Mean OLS-based ERT was downshifted relative to the true and IRLS-based ERT (see also Figure 4). Low-pass filtering had little effect on this pattern, but did result in a smoother signal that more closely

resembled the true ERT. By reducing artifactual high-frequency fluctuations, low-pass filtering reduced variance, causing signals across trials to be more tightly clustered around the mean ERT. **[C]** Mean (±SEM) percentage of mean peri-event waveforms in error compared to true transient across 10 simulations. By reducing ERT variance, low-pass filtering reduced the incidence of Type 2 errors for both OLS and IRLS-based dF/F, but also increased the incidence of Type 3 errors for OLS-based analyses. **[D]** Cumulative percentage of mean peri-event waveforms in error across 10 simulations.

Figure S3. Results of simulations with different underlying signal parameters (simulation set 2 [3Hz low-passed]). **[A-B]** Example fitting of simulated isosbestic onto corresponding experimental signal using OLS versus IRLS regression (whole-session **[A]**, and section indicated by dashed box **[B]**). A modest but consequential difference in baseline is observed. **[C-F]** Mean (±95% CI) absolute residuals during **[C]** non-event baseline, **[D]** across events, **[E]** first event, and **[F]** last event, following OLS vs. IRLS (c = tuning constant) regressions and dF vs. dF/F calculations. *$p < .05$. **[G]** True vs. extracted peri-event signals within a sample simulation. Colored waveforms = signal across trials (first [blue] and last [red] bolded); black waveform = mean waveform across trials. Vertical dashed lines = start/end of event; horizontal dashed line = session baseline. Waveform confidence interval (CI) analysis and periods of statistical error displayed beneath each panel. The true 8sec transient (leftmost panel) was constant across the session. OLS-based transients were downshifted, causing more misidentification of peri-event dynamics than IRLS-based transients. More change in signal across trials (spurious) is observed for dF-based compared to dF/F-based transients. **[H]** Mean (±SEM) percentage of waveform in error compared to true transient across 10 simulations. **[I]** Cumulative percentage of mean peri-

event waveforms in error across 10 simulations. OLS-based transients had more inferential errors than IRLS-based transients.

Figure S4. Comparison of recommended low-passed IRLS-based dF/F signals vs. detrended dF signals obtained via an established alternative analysis pipeline[11] for 1st (**[A-D]**) and 2nd (**[E-H]**) simulation sets. **[A,E]** Mean (±95% CI) absolute residuals for artifact-corrected signals during non-event (baseline; first panel), all events (second panel), first event (third panel), and last event (last panel) periods across 10 simulations. $^*p < .05$. **[B,F]** True vs. extracted peri-event signals within a sample simulation. Colored waveforms = signal across trials (first [blue] and last [red] bolded); black waveform = mean waveform across trials. Vertical dashed lines = start/end of event; horizontal dashed line = session baseline. Waveform confidence interval (CI) analysis and periods of statistical error displayed beneath each panel. Across simulation sets, detrended dF transients (middle panel) were downshifted relative to the true (left panel) and IRLS dF/F transients (right panel). A spurious change in signals across trials was more evident for detrended dF than IRLS dF/F. **[C,G]** Mean (±SEM) percentage of waveform in error compared to true transient across 10 simulations. **[D,H]** Cumulative percentage of mean peri-event waveforms in error across 10 simulations. Detrended dF had more inferential errors than IRLS dF/F transients.
